# Clinical Decision Support System for All Stages of Gastric Carcinogenesis in Real-Time Endoscopy: Model Establishment and Validation Study

**DOI:** 10.2196/50448

**Published:** 2023-10-30

**Authors:** Eun Jeong Gong, Chang Seok Bang, Jae Jun Lee, Hae Min Jeong, Gwang Ho Baik, Jae Hoon Jeong, Sigmund Dick, Gi Hun Lee

**Affiliations:** 1 Department of Internal Medicine Hallym University College of Medicine Chuncheon Republic of Korea; 2 Institute for Liver and Digestive Diseases Hallym University Chuncheon Republic of Korea; 3 Institute of New Frontier Research Hallym University College of Medicine Chuncheon Republic of Korea; 4 Department of Anesthesiology Hallym University College of Medicine Chuncheon Republic of Korea; 5 AIdot Seoul Republic of Korea

**Keywords:** atrophy, intestinal metaplasia, metaplasia, deep learning, endoscopy, gastric neoplasms, neoplasm, neoplasms, internal medicine, cancer, oncology, decision support, real time, gastrointestinal, gastric, intestinal, machine learning, clinical decision support system, CDSS, computer aided, diagnosis, diagnostic, carcinogenesis

## Abstract

**Background:**

Our research group previously established a deep-learning–based clinical decision support system (CDSS) for real-time endoscopy-based detection and classification of gastric neoplasms. However, preneoplastic conditions, such as atrophy and intestinal metaplasia (IM) were not taken into account, and there is no established model that classifies all stages of gastric carcinogenesis.

**Objective:**

This study aims to build and validate a CDSS for real-time endoscopy for all stages of gastric carcinogenesis, including atrophy and IM.

**Methods:**

A total of 11,868 endoscopic images were used for training and internal testing. The primary outcomes were lesion classification accuracy (6 classes: advanced gastric cancer, early gastric cancer, dysplasia, atrophy, IM, and normal) and atrophy and IM lesion segmentation rates for the segmentation model. The following tests were carried out to validate the performance of lesion classification accuracy: (1) external testing using 1282 images from another institution and (2) evaluation of the classification accuracy of atrophy and IM in real-world procedures in a prospective manner. To estimate the clinical utility, 2 experienced endoscopists were invited to perform a blind test with the same data set. A CDSS was constructed by combining the established 6-class lesion classification model and the preneoplastic lesion segmentation model with the previously established lesion detection model.

**Results:**

The overall lesion classification accuracy (95% CI) was 90.3% (89%-91.6%) in the internal test. For the performance validation, the CDSS achieved 85.3% (83.4%-97.2%) overall accuracy. The per-class external test accuracies for atrophy and IM were 95.3% (92.6%-98%) and 89.3% (85.4%-93.2%), respectively. CDSS-assisted endoscopy showed an accuracy of 92.1% (88.8%-95.4%) for atrophy and 95.5% (92%-99%) for IM in the real-world application of 522 consecutive screening endoscopies. There was no significant difference in the overall accuracy between the invited endoscopists and established CDSS in the prospective real-clinic evaluation (*P*=.23). The CDSS demonstrated a segmentation rate of 93.4% (95% CI 92.4%-94.4%) for atrophy or IM lesion segmentation in the internal testing.

**Conclusions:**

The CDSS achieved high performance in terms of computer-aided diagnosis of all stages of gastric carcinogenesis and demonstrated real-world application potential.

## Introduction

*Helicobacter pylori* is involved in and induces the early stages of gastric carcinogenesis, which begin with chronic gastritis and progress to atrophy, intestinal metaplasia (IM), and the development of gastric neoplasms [1,2]. Although *H. pylori* eradication reduces the risk of developing gastric cancer, the risk persists even after eradication, particularly in patients with advanced atrophy or IM in the stomach [3,4]. The primary goal of screening endoscopy is to detect neoplastic lesions in the stomach. However, preneoplastic conditions, such as atrophy and IM, require regular monitoring to detect developing neoplastic lesions [5,6]. Evidence for the recommended screening endoscopy time interval is lacking; however, a shorter interval is generally recommended for patients with atrophy or IM compared to those without [5-7]. Despite the importance of preneoplastic lesions in the stomach, general gastric cancer screening rather than individualized risk stratification has been used in Korea, where the prevalence of gastric cancer is highest [7].

Previously, our research group developed deep-learning–based automatic lesion detection and classification models for gastric neoplasms [8,9]. We improved the performance of these models by combining detection and classification models into a single clinical decision support system (CDSS) [10,11]. This CDSS was evaluated to see if it could add clinical benefit and show potential for real-world application. However, all of our previous research concentrated on gastric neoplasms, such as dysplasia, early gastric cancer (EGC), and advanced gastric cancer (AGC) [8-11]. Given that preneoplastic conditions are a risk factor for the development of gastric neoplasms, these lesions require attention as well, and more specific examinations for atrophy or IM are required.

Deep-learning–based computer-aided diagnosis models have been used in clinical practice, primarily to detect polyps in colonoscopies [12]. The most important advantages of these models are that they not only improve clinical performance but also reduce the burden on endoscopists from repetitive procedures and allow for greater concentration of professional activities [13]. These models also provide consistent and robust answers to physicians regardless of user fatigue [14-16]. Our previously established CDSS provided endoscopists with automated lesion detection and automated lesion classification functions [11]. The aim of these functions was to supplement the imperfect visual diagnosis by endoscopists and reduce the chances of missing important lesions. The primary advantage of this CDSS was to present robust answers to endoscopists regardless of the quantity of procedures, and the automated lesion detection function reduces the likelihood that significant lesions will be missed during endoscopic screening. All of these functions, however, were primarily focused on neoplastic lesions and neglected the preneoplastic condition.

Despite the importance of atrophy and IM, these stages were not considered in our previous study. Furthermore, there is no established model that categorizes all stages of gastric carcinogenesis. Models that only identify or diagnose IM and atrophy do not accurately represent real-world practice. As proposed by the Correa hypothesis [1], it is not known which patients with chronic gastritis, atrophy, IM, neoplasms, or gastric cancer will be tested in real-world practice. This study aimed to build and validate a CDSS in real-time endoscopy for all stages of gastric carcinogenesis, including atrophy and IM.

## Methods

### Ethical Considerations

This study was approved by the institutional review board of Hallym University Chuncheon Sacred Heart hospital (approval number: 2022-03-002). This study adhered to the guidelines for developing and reporting machine learning predictive models in biomedical research [17].

### General Concept

**Figure 1:**
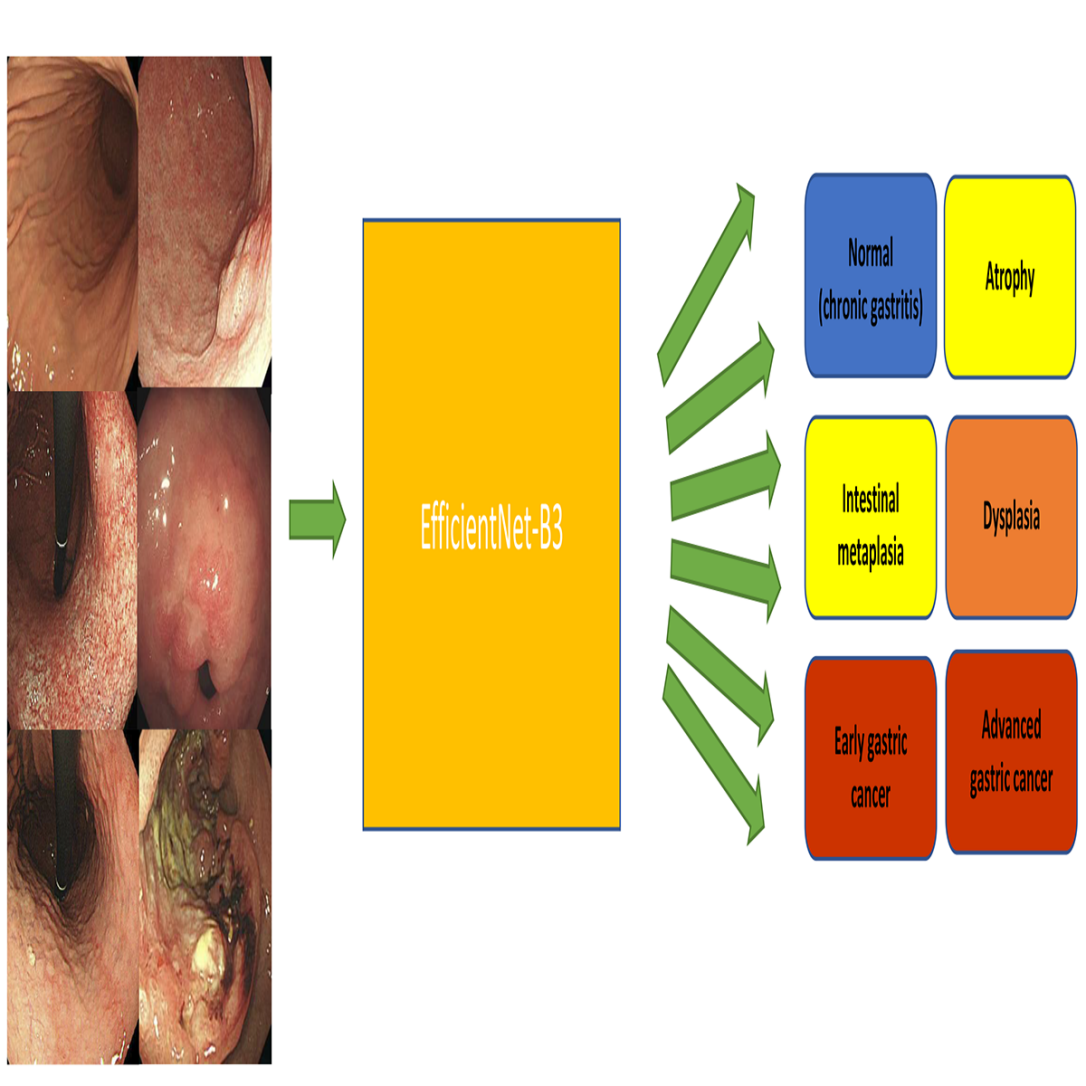
Schematic diagram of the establishment of the 6-class lesion classification model.

**Figure 2:**
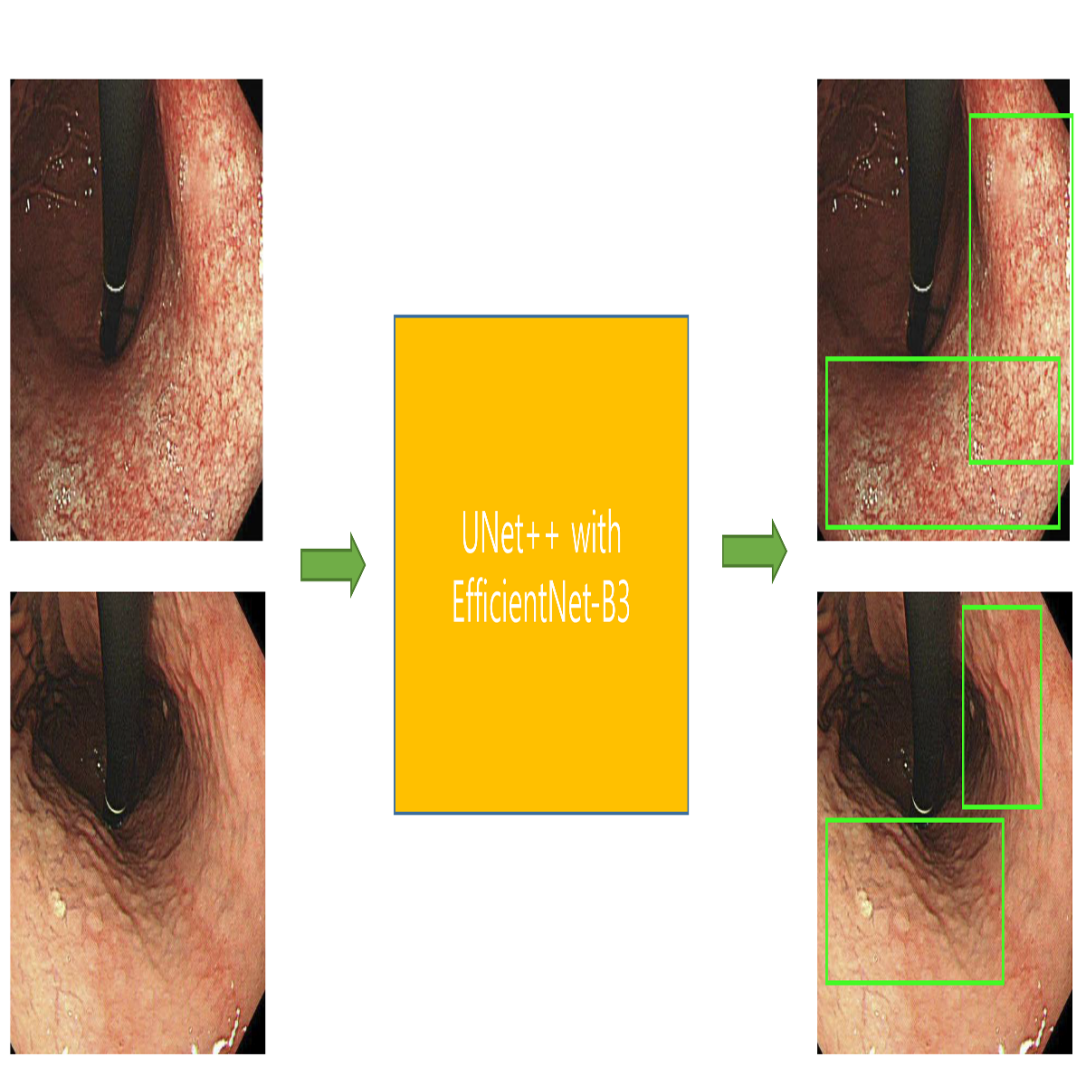
Schematic diagram of the establishment of the lesion segmentation model. Upper panels: segmentation of gastric atrophy. Lower panels: segmentation of intestinal metaplasia.

This study extends the previous research [8,9] on this topic by constructing a 6-class lesion classification model (Figure 1) and a preneoplastic lesion segmentation model (Figure 2). A CDSS was constructed by combining the established 6-class lesion classification model and the preneoplastic lesion segmentation model with the previously established lesion detection model. All images in the training, internal test, and external test data sets were mutually exclusive.

### Construction of Data Sets

We extended the data collection process in order to develop the automated lesion classification and lesion segmentation models. The CDSS was constructed using input images that were collected retrospectively. The detailed data collection procedure was described previously [8,9]. In brief, between 2010 and 2017, we enrolled consecutive patients with any type of gastric neoplasm discovered during upper gastrointestinal endoscopy and pathologically confirmed at the Chuncheon Sacred Heart hospital.

We enrolled all patients diagnosed with atrophy or IM during upper gastrointestinal endoscopy at Hallym University Chuncheon Sacred Heart hospital between 2019 and 2021 to add preneoplastic conditions to the baseline input data. These images were classified as either atrophy or IM. To reduce interobserver variability and ensure accurate categorization, all enrolled images were cross-checked by 2 expert endoscopists (CSB and EJG). Discordantly categorized images were resolved through discussion. The “normal” category was created using the same procedure as described above but without atrophy or IM. Endoscopic images of patients found to be free of gastric neoplasm, atrophy, or IM during upper gastrointestinal endoscopy at Hallym University Chuncheon Sacred Heart hospital between 2019 and 2021 were collected in JPEG format, with a minimum resolution of 640 x 480 pixels, from the in-hospital database [8,9].

Finally, 11,868 white-light images were enrolled and randomly divided into training (n=9999) and internal test (n=1869) data sets. Table 1 describes the detailed distribution of the input images.

**Table 1:** Data distribution for the establishment and testing of the clinical decision support system.

	Whole data set (N=13,150), n (%)	Training data set (n=9999), n (%)	Internal testing data set (n=1869), n (%)	External testing data set (n=1282), n (%)	Prospective real-clinic evaluation data set (n=522), n (%)
Advanced gastric cancer	840 (6.4)	524 (5.2)	130 (7)	186 (14.5)	3 (0.6)
Early gastric cancer	1257 (9.6)	842 (8.4)	225 (12)	190 (14.8)	4 (0.8)
Dysplasia	1115 (8.5)	735 (7.4)	197 (10.5)	183 (14.3)	8 (1.5)
Atrophy	2846 (21.6)	2228 (22.3)	375 (20.1)	243 (19)	252 (48.3)
Intestinal metaplasia	3857 (29.3)	3106 (31.1)	516 (27.6)	235 (18.3)	134 (25.7)
Normal	3235 (24.6)	2564 (25.6)	426 (22.8)	245 (19.1)	121 (23.2)

### Training Data Set Preparation

Endoscopic still-cut images contain “noise” information, such as the date of the examination and the patient’s name, age, gender, or identification number. The semantic segmentation of the input images can remove this noise information from the images and improve the training efficacy in the establishment of deep-learning models. For the preprocessing of the included images, we used a modified U-Net++ [18] convolutional neural network model (an edge-smoothing algorithm was added in the U-Net++, and the backbone convolutional neural network was Densenet121), as previously described [11]. Data augmentation methods were used, including rotation (–10° to +10°), horizontal or vertical flipping of included images, and image normalization with linear transformation in terms of the red, green, and blue channels [11].

### Deep-Learning Model Construction

All deep-learning models were built with Python (version 3.10; Python Software Foundation) and the SQLite 3.41.2 database management system (SQLite). The convolutional neural network models were implemented using the TensorFlow framework [19]. EfficientNet-B3 [20] was used to create the 6-class lesion classification model, while UNet++ with EfficientNet-B3 was used to create the lesion segmentation model. Additionally, 4 RTX 3090ti graphics processing units (MSI), AMD Ryzen Threadripper PRO 5975WX 32-Core central processing units, and 512 GB RAM (Samsung) were included in the training system.

### Development of the CDSS

**Figure 3:**
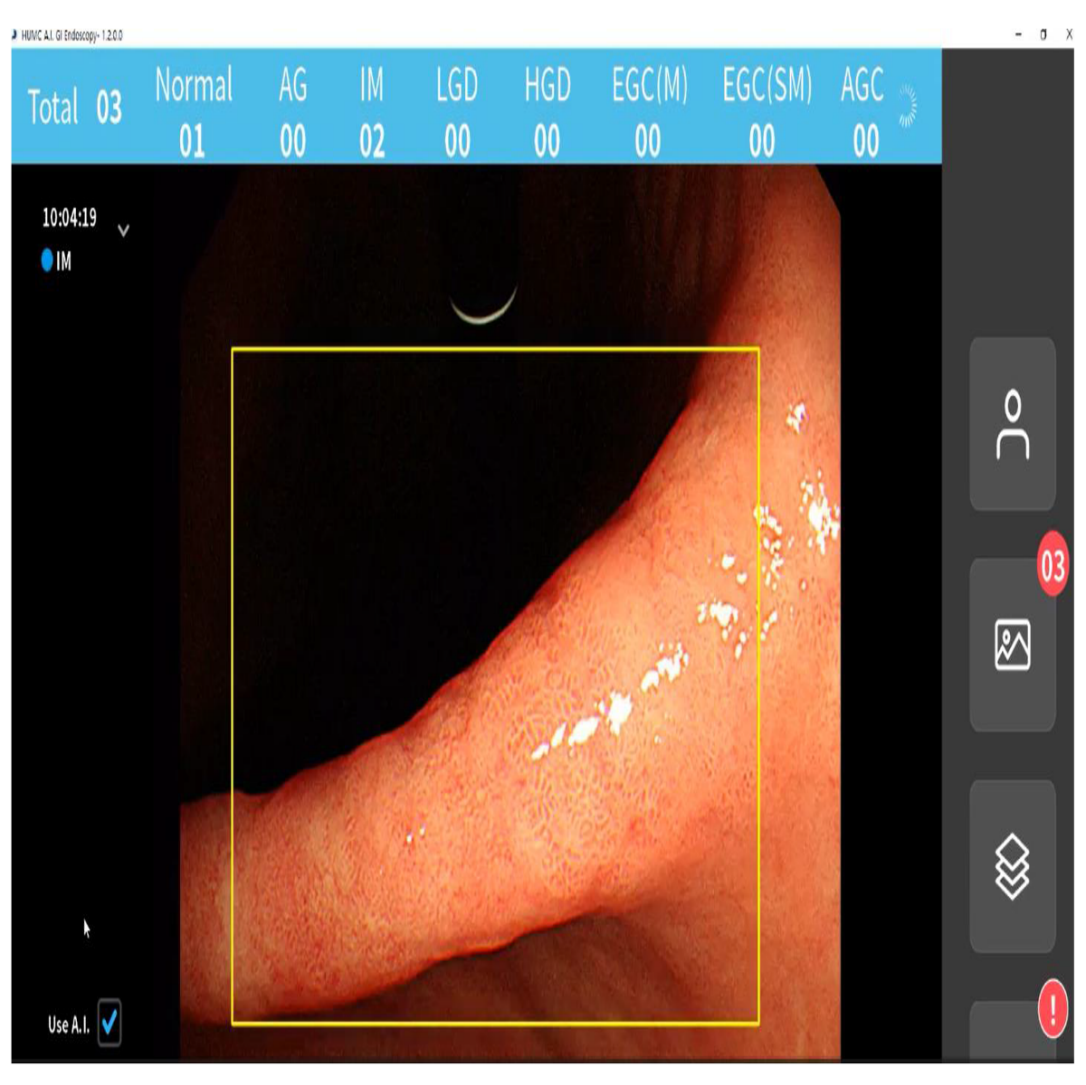
The established clinical decision support system's user interface. AG: atrophic gastritis; AGC: advanced gastric cancer; EGC(M): mucosa-confined early gastric cancer; EGC(SM): submucosa-invaded early gastric cancer; HGD: high-grade dysplasia; IM: intestinal metaplasia; LGD: low-grade dysplasia.

In order to maintain a seamless evaluation of the gastric mucosa without interfering with the endoscopic inspection, as previously explained, the CDSS was designed to give endoscopists additional information [11]. The primary deep-learning server and local computer system were first built independently. We linked the primary endoscopic system to a local computer with an additional display. The endoscope monitor image was entered into this local computer system, which then executed real-time detection tasks, asked the deep-learning server to classify the freeze images (called the motion freeze function), and displayed the results. The segmentation results were shown on the monitor after the atrophy or IM was identified and classified. The user interface for the established CDSS is described in Figure 3.

**Figure 4:**
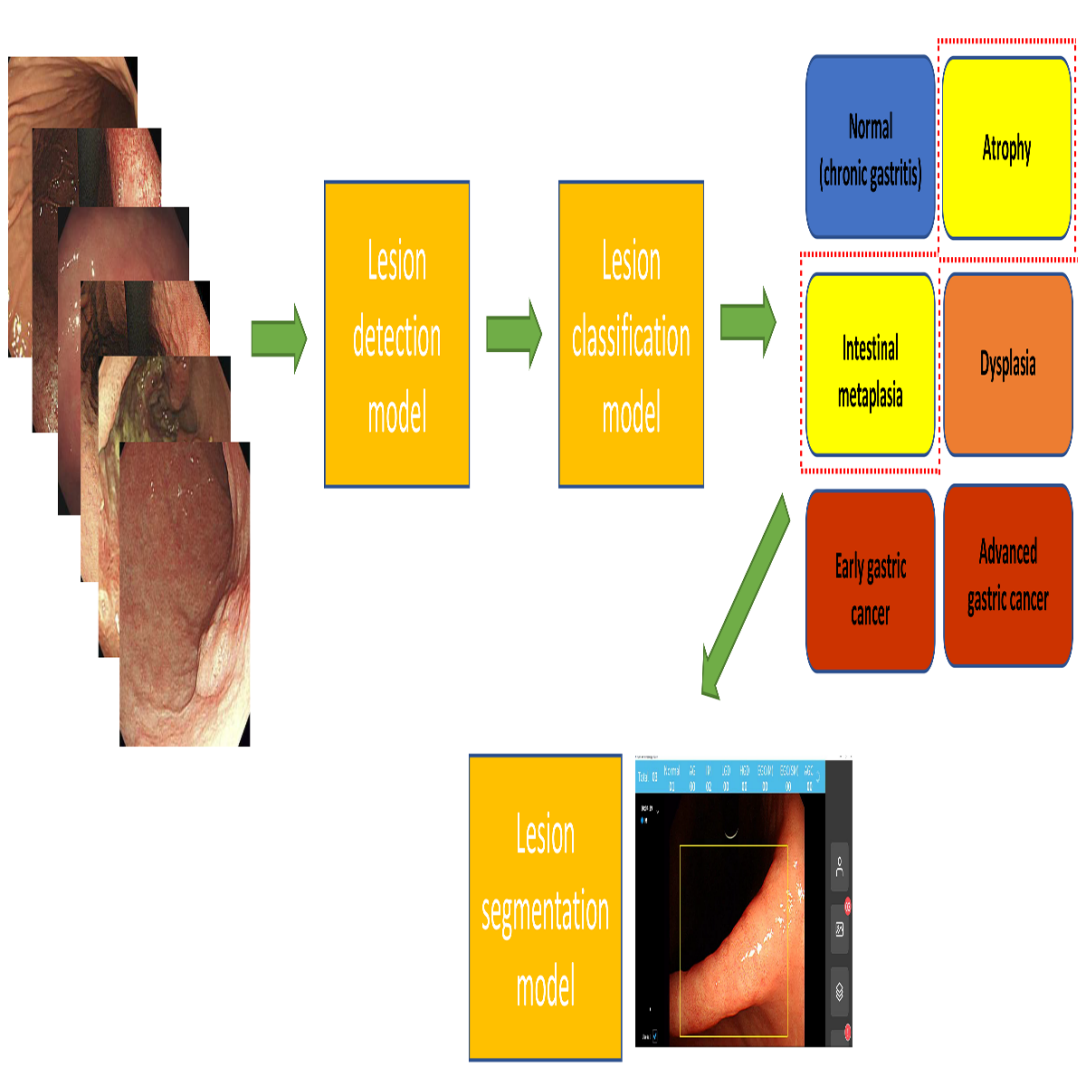
Schematic diagram of the establishment of clinical decision support system.

This study used a previously established lesion detection model [11]. Briefly, YOLOv3 [21], which was modified with EfficientNet-B0 structure, was implemented for the establishment of the model, and 2653 white-light images were enrolled for the training data set [11]. The detection model immediately transfers the detected gastric lesions to the classification model, where they are automatically classified into 6 classes (histologic diagnosis). When the CDSS detects a lesion, the frame containing the lesion is automatically examined by the classification model, yielding findings for 6 classes. The segmentation results are shown on the monitor after the atrophy or IM has been identified and classified (the detected and classified atrophy and IM are the input data of the segmentation model; Figure 4) The CDSS analyzes the dependency of successive frames (calculating the difference of brightness and contrast in successive frames) in order to detect the doctor’s intention (motion freeze function). The mean response time was 0.3 seconds between the classification request and the display of the findings on the monitor.

The video frame is encrypted and transferred with Academy Research Institute Agency encryption, which is a common cryptographic method by the Korean Agency for Technology and Standards used when a local computer requests a classification task from the main server to ensure anonymization. In order to prevent leakage and forgery or modification, medical information is also communicated between the deep-learning server and the local computer using the HTTP secure communication protocol and the secure sockets layer security protocol.

### Primary Study Outcomes

The primary outcomes included the classification model’s lesion classification accuracy (6 classes: AGC, EGC, dysplasia, atrophy, IM, and normal) and atrophy and IM lesion segmentation rates for the segmentation model. Diagnostic accuracy was defined as true positive + true negative / (true positive + false positive + true negative + false negative). Mean intersection over union (IoU) was defined as (*A*∩*B*) / (*A*∪*B*), and mean dice coefficient (F1 score) was defined as (2 × |*A*∩*B*|) / (|*A*|+|*B*|), where *A* signifies the predicted set of pixels (predicted segmentation map) and *B* is the ground truth of the object to be found in the image. Segmentation rate was defined as true positive / (true positive + false negative). For a positive image, true positive was defined as an IoU of the artificial intelligence (AI) prediction result mask greater than the threshold (0.5) and false negative was defined as an IoU of the AI prediction result mask less than the threshold (0.5).

### Performance Validation of the Established CDSS

To validate the performance of lesion classification accuracy, the following tests were performed (Table 1):

1. An external test using 1282 images from another institution: External test set images were collected from consecutive patients who underwent upper gastrointestinal endoscopy at the Gangneung Asan Hospital from 2018 to 2020.
2. Prospective evaluation of the classification accuracy of atrophy and IM in real-world procedures: Between January 2023 and March 2023, 522 consecutive screening endoscopy examinations were evaluated to measure the diagnostic accuracy of CDSS-assisted screening endoscopy for the diagnosis of gastric atrophy and IM in real-world applications. The expert endoscopist (CSB) performed all endoscopic examinations, and the diagnostic accuracy rate for gastric atrophy and IM was measured.

To estimate the clinical utility of the CDSS, 2 additional experienced endoscopists were invited to perform a blind test with the same data set. To compare the diagnostic performances of the CDSS and endoscopists, we performed Fisher exact tests. In this study, a *P* value <.05 (2-tailed) was adopted as the threshold of statistical significance. The analyses were performed using SPSS version 24.0 (SPSS).

## Results

### Characteristics of the Data Set

A total of 13,150 endoscopic still-cut images were included in the study, comprising the training data set (n=9999), the internal test data set (n=1869), and the external test data set (n=1282). Of the images included in the training and internal test data sets (n=11,868), those with IM made up the largest proportion (n=3622; 30.5%), followed by normal (n=2990; 25.2%), atrophy (n=2603; 21.9%), EGC (n=1067; 9%), dysplasia (n=932; 7.9%), and AGC (n=654; 5.5%) images.

For the external data set, which comprised 1282 images collected from another institution, the proportion of normal images was the largest at 19.1% (n=245), followed by atrophy images (n=243; 19%), IM images (n=235; 18.3%), EGC images (n=190; 14.8%), AGC images (n=186; 14.5%), and dysplasia images (n=183; 14.3%). These proportions reflect the distinctive characteristics of the institution from which the images were collected.

A total of 522 consecutive screening endoscopy images were enrolled for prospective real-clinic evaluation of the established CDSS. The proportion of images with atrophy was the largest (n=252; 48.3%), followed by those with IM (n=134; 25.7%), dysplasia (n=8; 1.5%), EGC (n=4; 0.8%), and AGC (n=3; 0.6%). Details of the quantity of images and the distribution of categories are presented in Table 1.

### Internal Test Performance of CDSS Classification Accuracy

Multimedia Appendices 1 and 2 describe the schematic framework of the established lesion classification and lesion segmentation models. The overall 6-class lesion classification accuracy (95% CI) in the internal test data set of the classification models was 90.3% (89%-91.6%). The accuracy for each category was as follows: atrophy was 96.5% (94.9%-98.1%), IM was 90.4% (87.4%-93.4%), EGC was 78.2% (72.8%-83.6%), AGC was 86.2% (80.3%-92.1%), dysplasia was 75.6% (69.6%-81.6%), and normal was 97% (95.4%-98.6%). The detailed classification performance of the established CDSS is described in Tables 2 and 3, and a confusion matrix for the internal test classification performance is presented in Multimedia Appendix 3. The detailed hyperparameter information for the established lesion classification model was as follows: weight initialization with ImageNet; augmentation, horizontal/vertical flip, rotate (−10° to +10°); input size, 300 x 300; batch size, 32; learning rate, 1 × 10^-04^; epoch, 100; dropout, 0.2; optimizer, SGD; loss function, cross-entropy loss; and learning rate scheduler, cosine decay.

**Table 2:** Summary of the accuracy of internal and external test classifications by the established clinical decision support system.

Class	Internal test data set (n=1869), % (95% CI)	External test data set (n=1282), % (95% CI)	Prospective real-clinic evaluation data set (n=522), % (95% CI)
Overall	90.3 (89-91.6)	85.3 (83.4-97.2)	89.3 (86.6-92)
Advanced gastric cancer	86.2 (80.3-92.1)	82.8 (77.4-88.2)	100 (100-100)
Early gastric cancer	78.2 (72.8-83.6)	76.3 (70.3-82.3)	50 (1-99)
Dysplasia	75.6 (69.6-81.6)	74.9 (68.6-81.2)	75 (45-100)
Atrophy	96.5 (94.9-98.1)	95.3 (92.6-98)	92.1 (88.8-95.4)
Intestinal metaplasia	90.4 (87.4-93.4)	89.3 (85.4-93.2)	95.5 (92-99)
Normal	97 (95.4-98.6)	88.2 (84.2-92.2)	78.5 (71.2-85.8)

**Table 3:** Summary of the per-class classification performance of the established clinical decision support system for the internal and external test data sets.

		Accuracy	Precision	Recall	F1 score
**Internal test data set, % (95% CI)**					
	Advanced gastric cancer	86.2 (80.3-92.1)	88.9 (83.5-94.3)	86.2 (80.3-92.1)	87.5 (81.8-93.2)
	Early gastric cancer	78.2 (72.8-83.6)	81.1 (76-86.2)	78.2 (72.8-83.6)	79.6 (74.3-84.9)
	Dysplasia	75.6 (69.6-81.6)	70.6 (64.2-77)	75.6 (69.6-81.6)	73.0 (66.8-79.2)
	Atrophy	96.5 (94.9-98.1)	94.1 (91.7-96.5)	96.5 (94.6-98.4)	95.3 (93.2-97.4)
	Intestinal metaplasia	90.4 (87.4-93.4)	91.4 (89-93.8)	90.4 (87.4-93.4)	90.9 (88.4-93.4)
	Normal	97 (95.4-98.6)	99.5 (98.8-99.9)	97 (95.4-98.6)	98.2 (96.9-99.5)
**External test data set, % (95% CI)**					
	Advanced gastric cancer	82.8 (77.4-88.2)	95.7 (92.8-98.6)	82.8 (77.4-88.2)	88.8 (84.3-93.3)
	Early gastric cancer	76.3 (70.3-82.3)	81.9 (76.4-87.4)	76.3 (70.3-82.3)	79 (73.2-84.8)
	Dysplasia	74.9 (68.6-81.2)	76.5 (70.4-82.6)	74.9 (68.6-81.2)	75.7 (69.5-81.9)
	Atrophy	95.3 (92.6-98.0)	80.9 (76-85.8)	95.3 (92.6-98)	87.5 (83.3-91.7)
	Intestinal metaplasia	89.3 (85.4-93.2)	85.8 (81.3-90.3)	89.3 (85.3-93.3)	87.5 (83.3-91.7)
	Normal	88.2 (84.2-92.2)	91.9 (88.5-95.4)	88.2 (84.2-92.2)	90 (86.2-93.8)

In terms of the lesion segmentation rate, the CDSS demonstrated a segmentation rate of 93.4% (95% CI 92.4%-94.4%) for atrophy or IM lesion segmentation in the internal test data set (Table 4). Multimedia Appendix 4 shows the representative segmentation results of atrophy and IM locations that correspond to the ground truth area. The detailed hyperparameter information for the established lesion segmentation model was as follows: weight initialization with ImageNet; augmentation, horizontal/vertical flip, rotate (−10° to +10°); input size, 352 x 352; batch size, 16; learning rate, 1 × 10^-04^; epoch, 100; dropout, 0.2; optimizer, Adam; loss function, binary cross-entropy + IoU; and weight decay, 1 × 10^-04^.

**Table 4:** Summary of the segmentation performance of the established clinical decision support system.

	IoU^a^ (%), mean (SD)			Dice (%), mean (SD)			Segmentation rate, % (95% CI)
	Atrophy	IM^b^	Total	Atrophy	IM	Total	
Validation	79.2 (1.6)	66.6 (1.9)	75 (1.7)	87.6 (1.1)	77.7 (1.8)	84.7 (1.4)	93.4 (92.4-94.4)
Internal test	79.4 (1.5)	67.6 (1.9)	75.3 (1.7)	87.8 (1.0)	78.7 (1.7)	84.9 (1.3)	93.4 (92.4-94.4)

^a^IoU: intersection over union.

^b^IM: intestinal metaplasia.

### Performance Validation for the 6-Class Lesion Classification Model

For performance validation with the external test data set, the CDSS achieved an overall accuracy (95% CI) of 85.3% (83.4%-97.2%). The accuracy for each category was as follows: atrophy was 95.3% (92.6%-98%), IM was 89.3% (85.4%-93.2%), EGC was 76.3% (70.3%-82.3%), AGC was 82.8% (77.4%-88.2%), dysplasia was 74.9% (68.6%-81.2%), and normal was 88.2% (84.2%-92.2%). The detailed performance of the established CDSS is described in Table 2, and a confusion matrix for the external test performance is presented in Multimedia Appendix 5.

For performance validation with the prospective real-clinic evaluation data set, the CDSS achieved an overall accuracy of 89.3% (86.6%-92%). The accuracy for each category was as follows: atrophy was 92.1% (88.8%-95.4%), IM was 95.5% (92%-99%), EGC was 50% (1%-99%), AGC was 100% (100%-100%), dysplasia was 75% (45%-100%), and normal was 78.5% (71.2%-85.8%). The detailed performance of the established CDSS is described in Table 2**,** and a confusion matrix for the prospective real-clinic evaluation performance is presented in Multimedia Appendix 6.

To estimate the clinical utility of the CDSS, 2 experienced endoscopists (HMJ and GHB) were invited to perform a blind test with the same data set. For the first endoscopist, the overall accuracy was 87.5% (84.7%-90.3%), and the accuracy for each category was as follows: atrophy was 86.1% (81.8%-90.4%), IM was 80.6% (73.9%-87.3%), EGC was 75% (32.6%-99%), AGC was 100% (100%-100%), dysplasia was 62.5% (29%-96%), and normal was 100% (100%-100%). For the second endoscopist, the overall accuracy was 90.8% (88.3%-93.3%), and the accuracy for each category was as follows: atrophy was 91.3% (87.8%-94.9%), IM was 84.4% (73.9%-94.8%), EGC was 100% (100%-100%), AGC was 100% (100%-100%), dysplasia was 87.5% (64.6%-100%), and normal was 90.9% (85.8%-96%). The Cohen κ for the interrater reliability between the 2 endoscopists was 0.83. There was no significant difference in the overall accuracy between the invited endoscopists and the established CDSS in the prospective real-clinic evaluation data set (*P*=.23).

### Representative Examples of the CDSS for Real-Clinic Application

Multimedia Appendix 7 shows representative examples of the CDSS for the classification and segmentation of gastric atrophy and IM. The established CDSS detected, classified, and segmented gastric atrophy and IM on the angle and lesser curvature sides of the body. A small subepithelial tumor was detected and correctly classified as normal.

## Discussion

### Principal Findings

This study established a CDSS that accurately classifies all stages of gastric carcinogenesis and confirmed that the diagnostic accuracy of 6 classes is potentially accurate for real-world clinical applications. Our research group previously established a classification model with 4 or 5 classes for the diagnosis of detected lesions during upper gastrointestinal endoscopy [8,11]. Normal, dysplasia (low-grade or high-grade), EGC, and AGC were the diagnostic categories. However, preneoplastic conditions, such as gastric atrophy and IM, are important categories in which hidden neoplastic lesions or potential lesions for the development of neoplastic lesions are implicated. Our previously established CDSS did not include these categories and considered them to be normal [8,11]. In general, as the number of target classification categories increases, classification accuracy decreases. However, when compared to our other models, our established CDSS performed similarly or even better in terms of diagnostic performance. This is most likely due to an increase in the number of training data images.

### Comparison to Prior Work

The presence of external validation is one of the strengths of this study. Even the majority of US Food and Drug Administration–approved medical AI models lack external validation, which ensures the established model’s generalizability [22]. The majority of currently established models are trained on data from their own institution. As a result, internal test performance is good; however, external test performance suffers due to the “data set shift” phenomenon (differences in data settings, patient characteristics, and so on) [14-17,22]. Our established model also demonstrated lower external test performance when compared to internal test performance; however, the magnitude of the performance decrease was minor, and external test accuracy was still high enough to potentially be used in real-clinic applications.

We also performed prospective real-clinic application performance validation to decrease the gap between theory and practice. When compared to the training data set, real-world clinical practice has distinct characteristics. For the training data set, we included an even number of images in each category; this was because class distribution influences the overall performance of the established model [16]. The inclusion of a class with an insufficient number of images negatively impacts the performance for that class and the established model as a whole; this is known as spectrum bias [14-16]. However, clinical practice differs. The majority of screening endoscopies are classified as “normal,” with only a small number of patients being diagnosed with neoplastic conditions. As a result, neoplastic lesion–focused AI diagnosis models may be clinically ineffective. The current CDSS was developed using the previously established 4-class diagnosis model of gastric neoplasms [11]. We added the ability to make more precise diagnoses of preneoplastic or nonneoplastic conditions. As a result, the current CDSS may be useful in real-world applications. Prospective real-clinic application performance revealed potentially high accuracy not only for gastric atrophy and IM lesions, but also overall accuracy. There was also no significant difference in the overall accuracy between the experienced endoscopists and the established CDSS.

Target conditions and training data sets distinguish our established CDSS from previously reported models. Guimarães et al [23] created an atrophy detection model that outperformed expert endoscopists with 93% accuracy in an independent data set. Zhang et al [24] also developed a lesion detection model for gastric atrophy that was 94% accurate. Zhao and Chi [25] also developed a gastric atrophy lesion detection model and demonstrated that using this model improved gastric atrophy diagnosis rates when compared to not using the model. Luo et al [26] created a lesion detection model for gastric atrophy that outperformed endoscopists in detection performance. However, the utility of this type of data set in real-world clinical practice is limited. These studies generated a training data set that included only atrophy and no atrophy categories. The categories of gastric cancer, normal, and IM were not considered in these studies. Because they only allow for discrimination between atrophy versus no atrophy, the clinical utility of these models is limited. Siripoppohn et al [27] developed a real-time semantic segmentation model for IM; despite its excellent diagnostic performance, only IM could be segmented in real time.

### Strengths

Various conditions are included in the “normal” category of upper gastrointestinal endoscopy, which excludes neoplastic lesions. It is common to have gastritis without atrophy or IM. These include mucosal edema or exudates, hemorrhagic spots, erosions, hyperemic mucosa, and other conditions. On the other hand, there are also more complicated benign conditions, such as benign ulcers or ulcer scars, subepithelial tumors, xanthomas, nonneoplastic polyps (eg, fundic gland or hyperplastic polyps), or angiodysplasias. To complement the endoscopic inspection without interfering with the operator’s examination, our CDSS was established. This CDSS detects stomach lesions automatically and provides additional information (histologic prediction) via an alarm sound and a lesion detection box. The effect of this system varies depending on the level of expertise of the endoscopist, and previous research has shown that endoscopists with a certain level of expertise benefit the most from this CDSS [10].

### Future Directions

Multimodal AI foundation models, including large language models, are becoming more popular. This type of model may perform a more sophisticated practice involving decision making. Using these models, it would be possible to create a multimodal CDSS capable of not only supplementing endoscopic examinations but also writing endoscopic reports and providing personalized explanations or recommendations [22]. CDSS detection and classification of all gastric lesions, including benign conditions, may not be required for expert endoscopists; however, trainee or novice endoscopists may require the assistance of this CDSS to ensure a consistent level of performance for the detection or classification of lesions during endoscopic examination. Creating such complex models would be possible with the help of multimodal foundation AI models.

### Limitations

Despite the benefits of our established CDSS, an inherent flaw arising from the study design and retrospectively collected data was discovered in this study. The training data set was obtained from a single institution, which may indicate a bias in selection or spectrum. Because of the unique characteristics of each institution’s patients, medical AI models developed from a single institution typically have limitations for widespread implementation, highlighting the importance of external testing. To compensate for this flaw, we used rigorous prospective validations using images from another institution. In addition, we validated the clinical utility through prospective real-clinic application validation.

In conclusion, the CDSS demonstrated high performance in terms of computer-aided diagnosis of all stages of gastric carcinogenesis and demonstrated real-world application potential.

## Appendix

Multimedia Appendix 1 Structure of the established lesion classification model (1). FC: fully connected layer; MBConv: mobile inverted bottleneck convolution.

Multimedia Appendix 2 Structure of the established lesion classification model (2). FC: fully connected layer; MBConv: mobile inverted bottleneck convolution; SE: squared exponential.

Multimedia Appendix 3 Confusion matrix for the internal test classification performance. AGC: advanced gastric cancer; EGC: early gastric cancer; IM, intestinal metaplasia.

Multimedia Appendix 4 Representative segmentation results of atrophy and IM locations that correspond to the ground truth area (original image vs model prediction vs ground truth).

Multimedia Appendix 5 Confusion matrix for the external test classification performance. AGC: advanced gastric cancer; EGC: early gastric cancer; IM: intestinal metaplasia.

Multimedia Appendix 6 Confusion matrix for the prospective real-clinic evaluation performance. AGC: advanced gastric cancer; EGC: early gastric cancer; IM: intestinal metaplasia.

Multimedia Appendix 7 Representative examples of the use of the clinical decision support system for the classification and segmentation of gastric atrophy and intestinal metaplasia.

## Abbreviations

AGC: advanced gastric cancer
CDSS: clinical decision support system
EGC: early gastric cancer
IM: intestinal metaplasia
IoU: intersection over union

## Data Availability

All the data are accessible and available from the corresponding author upon request.
