# Supplementary figures and images for: Clinical Decision Support System for All Stages of Gastric Carcinogenesis in Real-Time Endoscopy: Model Establishment and Validation Study
Source: J Med Internet Res. 2023 Oct 30;25:e50448. doi: 10.2196/50448 (PMC10644184; doi:10.2196/50448)

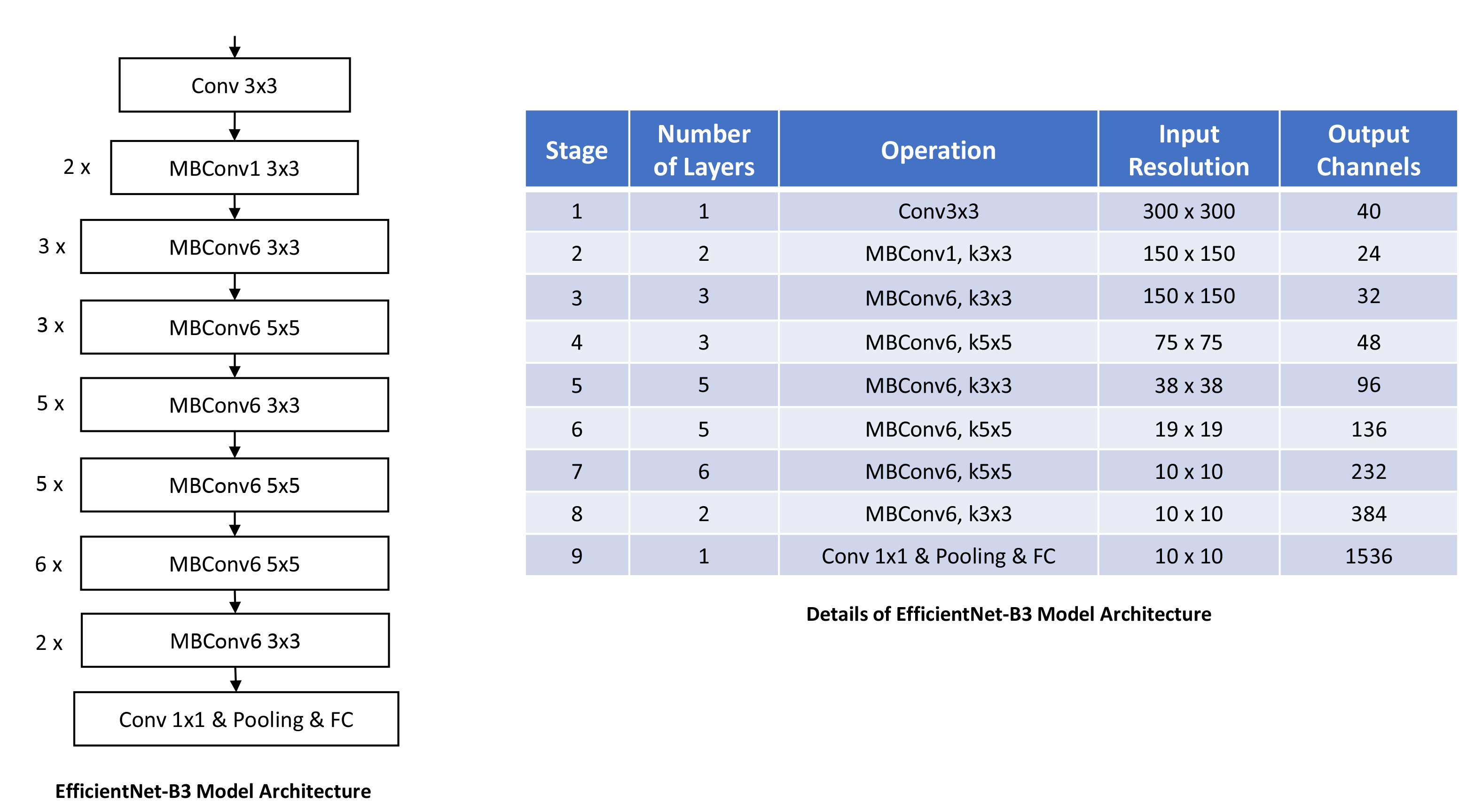

Supplement: Multimedia Appendix 1 [file jmir_v25i1e50448_app1.png]

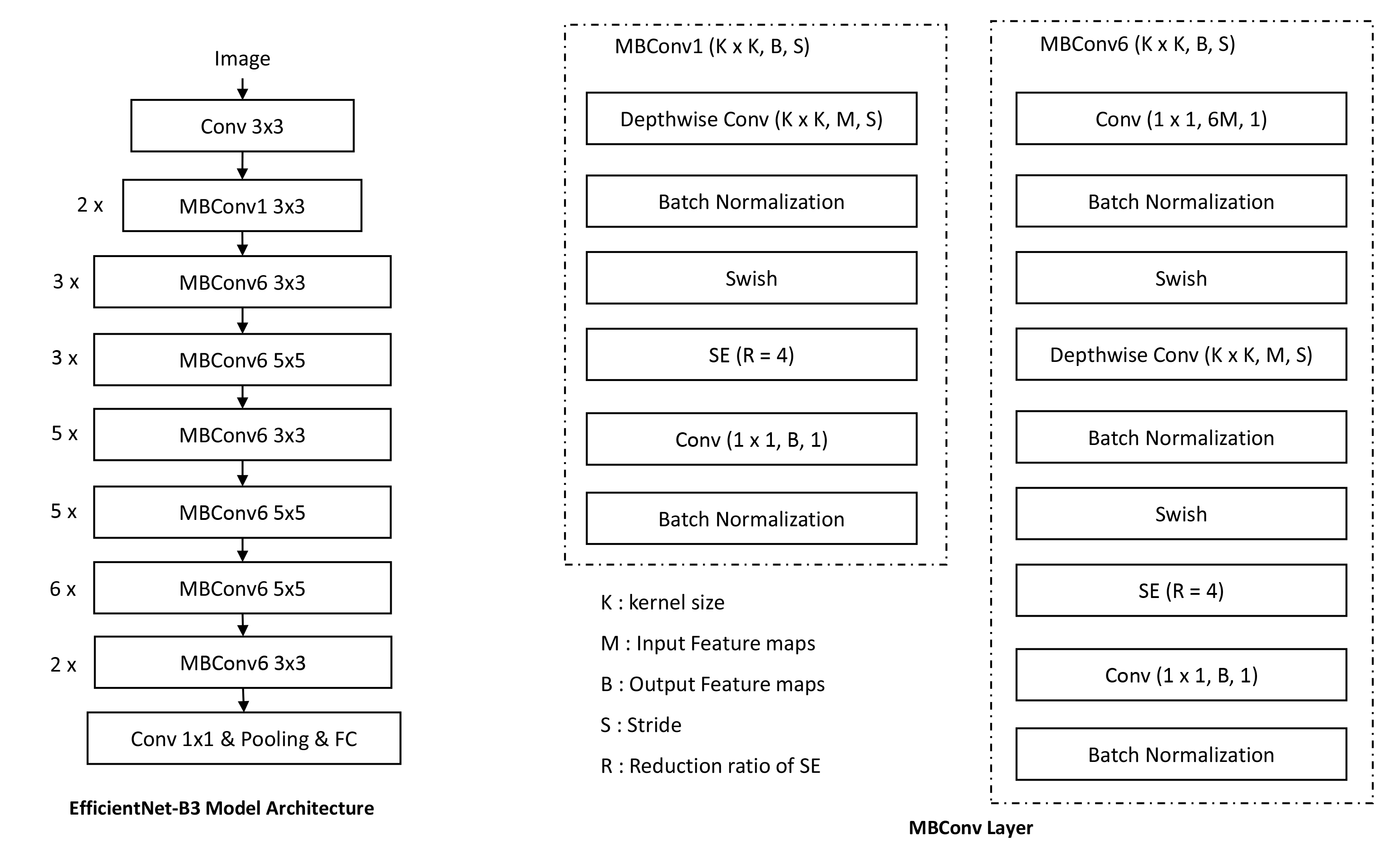

Supplement: Multimedia Appendix 2 [file jmir_v25i1e50448_app2.png]

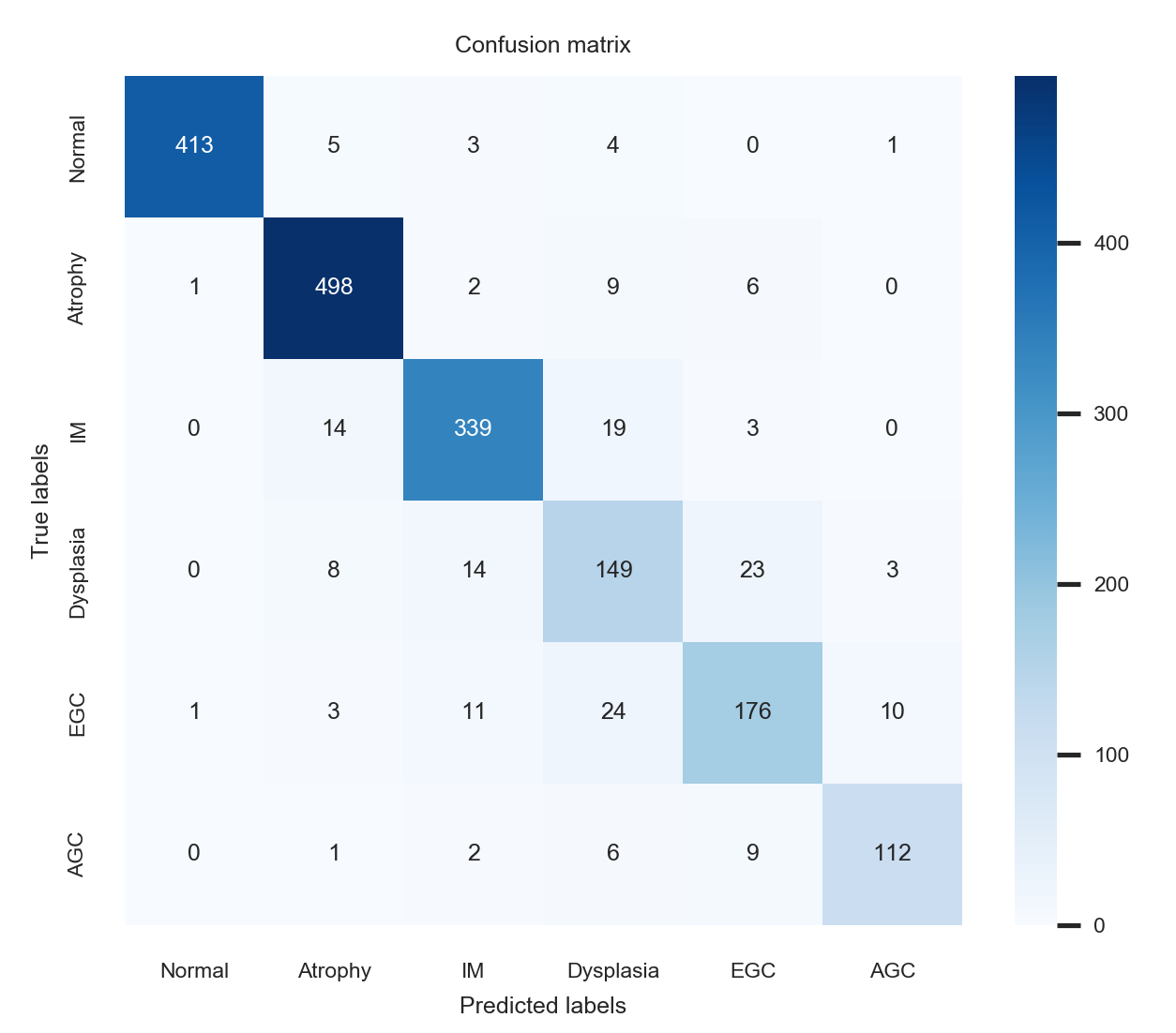

Supplement: Multimedia Appendix 3 [file jmir_v25i1e50448_app3.png]

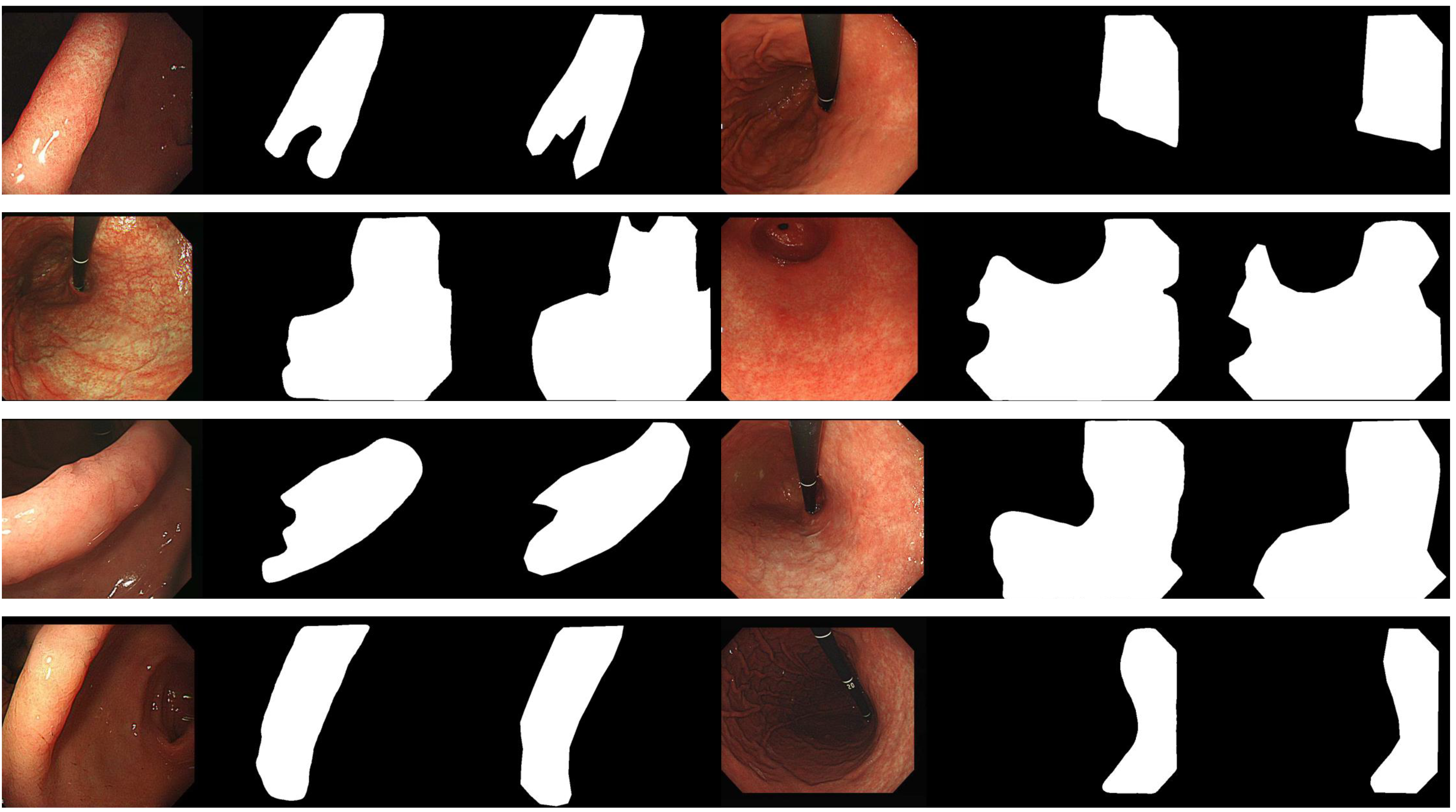

Supplement: Multimedia Appendix 4 [file jmir_v25i1e50448_app4.png]

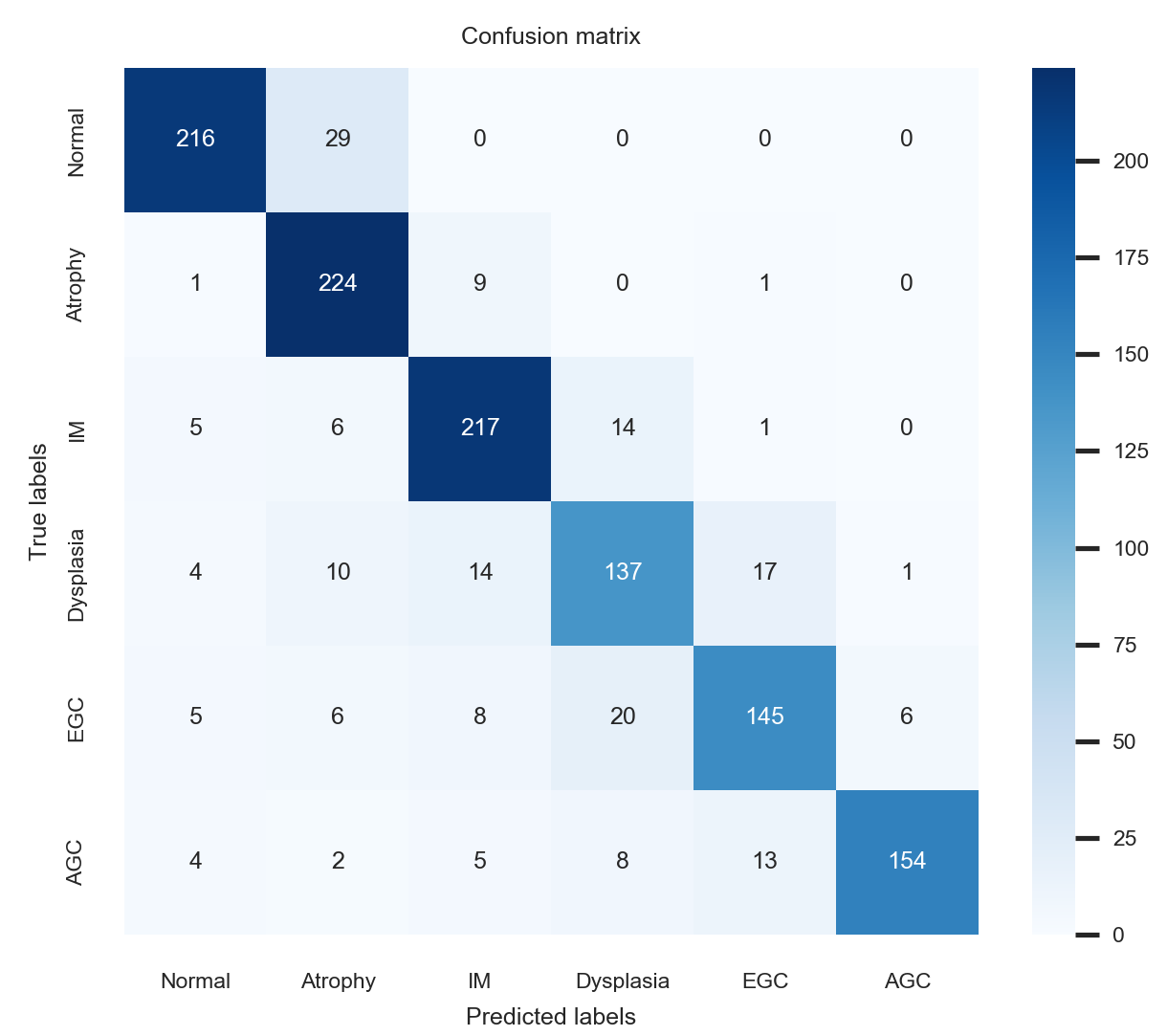

Supplement: Multimedia Appendix 5 [file jmir_v25i1e50448_app5.png]

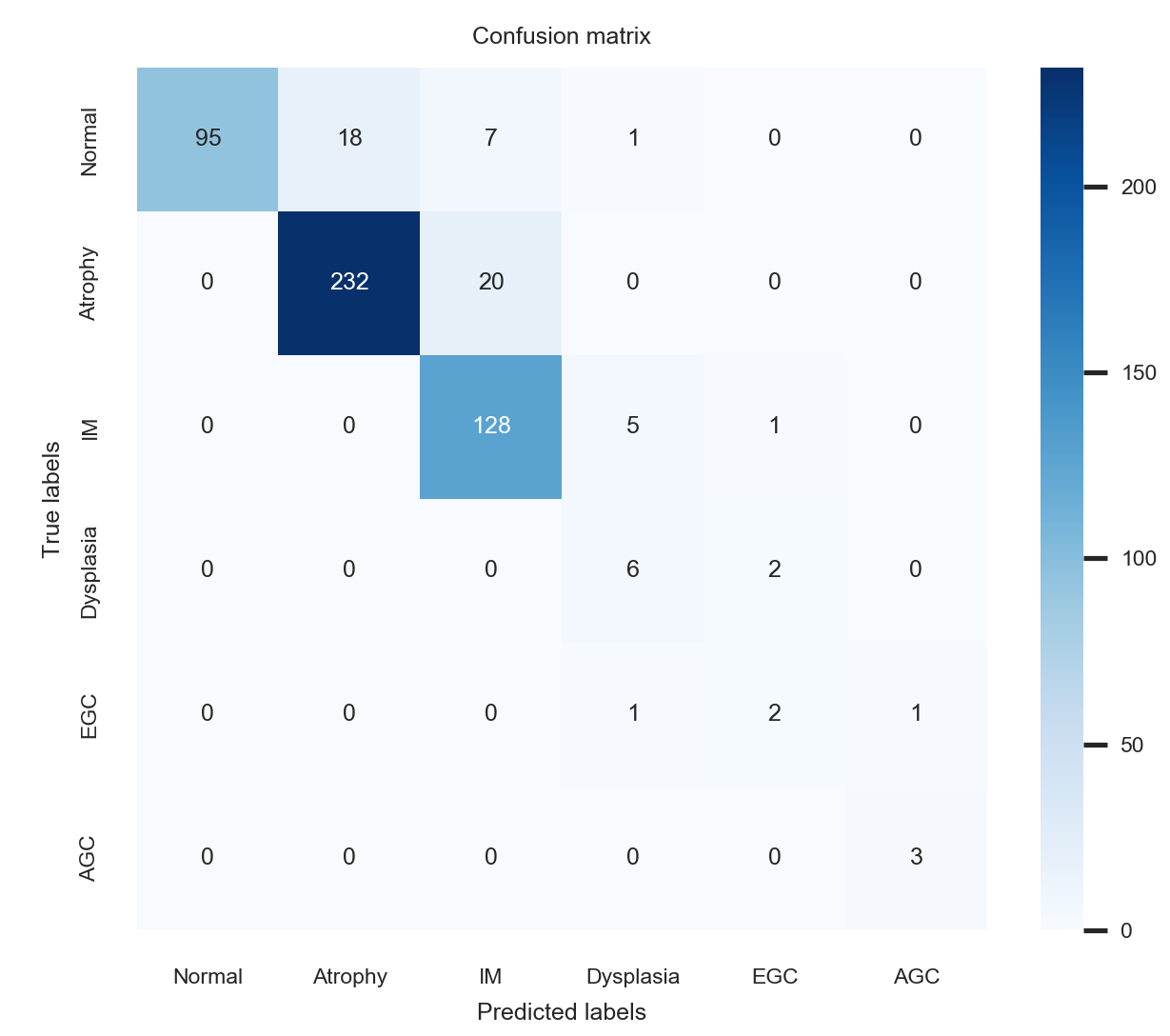

Supplement: Multimedia Appendix 6 [file jmir_v25i1e50448_app6.png]
